# Simultaneous Determination of Rutin, Luteolin, Quercetin, and Betulinic Acid in the Extract of* Disporopsis pernyi* (Hua) Diels by UPLC

**DOI:** 10.1155/2015/130873

**Published:** 2015-12-22

**Authors:** Yanqi Wang, Shuyi Li, Dandan Han, Kehan Meng, Miao Wang, Chunjie Zhao

**Affiliations:** ^1^School of Pharmacy, Shenyang Pharmaceutical University, Shenyang 110016, China; ^2^School of Life Science and Biopharmaceutics, Shenyang Pharmaceutical University, Shenyang 110016, China

## Abstract

*Disporopsis pernyi* (Hua) Diels, which belongs to genus* Disporopsis*, has been widely used for the treatment of abnormal sweating, chronic cough, and so forth. An ultra-performance liquid chromatography (UPLC) analysis was developed for the determination of rutin, luteolin, quercetin, and betulinic acid in* Disporopsis pernyi* (Hua) Diels roots. UPLC analysis was conducted by using a Shim-pack XR-ODS column with gradient elution with the mobile phase of acetonitrile and water containing 0.1% formic acid and with a flow rate of 0.2 mL/min, detected at 210, 254, and 280 nm. The method was precise, with relative standard deviation < 2.0%. The recoveries for the four components in* Disporopsis pernyi* (Hua) Diels were between 98.5 and 100.9%. The average contents of rutin, luteolin, quercetin, and betulinic acid in roots were 5.63, 2.51, 3.87, and 2.41 *μ*g/g, respectively. The method was accurate and reproducible and it can provide a quantitative basis for quality control of* Disporopsis pernyi* (Hua) Diels.

## 1. Introduction

*Disporopsis pernyi* (Hua) Diels, which belongs to* Disporopsis* genus of Liliaceae family, mainly grows in South Asia such as Vietnam, Laos, Thailand, and Yangtze river basin of China. It was a well-known traditional Chinese medicine which has been widely used for the treatment of abnormal sweating, chronic cough, women postpartum weakness and irregular menstrual cycle, and so forth [1]. The medical value of* Disporopsis* genus plants has not got much attention until the beginning of 21st century. The roots of the plant are rich in bioactive substances which are possible to have considerable medicinal value and can be used for hypertension cough, inflammation, and tumor. Study suggests that a total of 4 polyphenolic compounds are found in extract including rutin, luteolin, quercetin, and betulinic acid, and the phenolic compounds contribute significantly to the antioxidant and antimicrobial activities [2, 3]. Rutin has both antihypertensive effect and antidiabetes effect [4, 5]. Luteolin which has anti-inflammatory, antibiosis, and anticancer properties has been used for relieving cough and eliminating phlegm. In addition, it has potential anti-HIV effect [6, 7]. Quercetin can be used for relieving cough and eliminating phlegm and for hypertension and hyperlipemia; also, it has neuroprotective and antiproliferative activities [8, 9]. Betulinic acid can kill human melanoma cell without hurting healthy cell and inhibit the HIV-1 infection. Additionally, recent study shows that it also has the inhibition effects of cerebroma and leukocythemia [10, 11].

The four compounds are major bioactive constitutes in the extract of* Disporopsis pernyi* (Hua) Diels roots. So far, there is no report on the content of the 4 polyphenolic compounds in* Disporopsis pernyi* (Hua) Diels. Therefore, it is important to determine the content of the four components. The four components were quantified by ultra-performance liquid chromatography (UPLC). UPLC is a simple and quick tool for the quantitative determination of the bioactive constituents in pharmaceutical industry [12–14]. As rutin, luteolin, quercetin, and betulinic acid are strong chromophores, this makes UV detection easy and feasible [15–18].

## 2. Materials and Methods

### 2.1. Chemicals and Reagents

**Figure 1:**
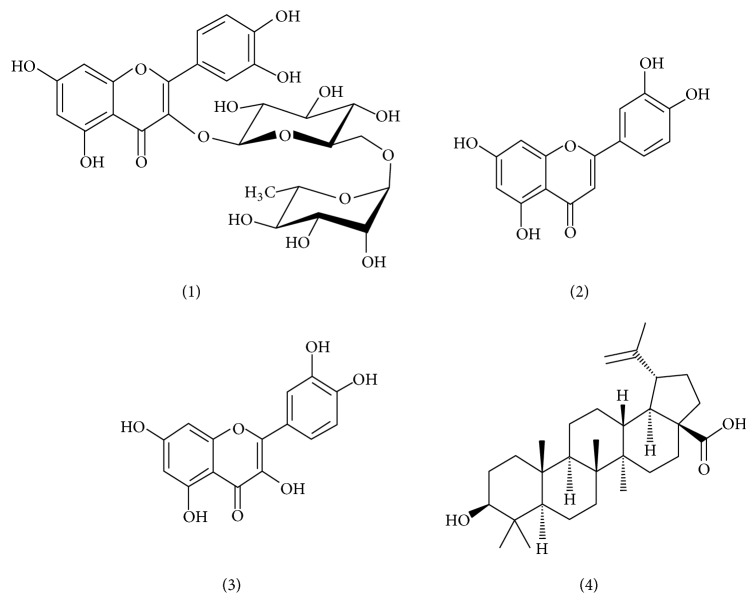
Chemical structures of compounds in* Disporopsis pernyi* (Hua) Diels. (1) Rutin, (2) luteolin, (3) quercetin, and (4) betulinic acid.

The HPLC-grade methanol and acetonitrile used were purchased from Caledon (Canada) and formic acid was obtained from Dima Company (Beijing, China). Water was purchased from Hangzhou Wahaha Company, China. The rutin, luteolin, quercetin, and betulinic acid were purchased from the National Institute for the Control of Pharmaceutical and Biological Products in China. The purity of the standard compounds was ≧98%; their chemical structures are shown in Figure 1.* Disporopsis pernyi* (Hua) Diels were collected from Songtao which is in Guizhou Province of China.

### 2.2. Preparation of Standard Stock Solutions and Sample Solution

Standard stock solutions of rutin, luteolin, quercetin, and betulinic acid were dissolved in methanol, at concentration of 1.0 mg/mL. All standard solutions were filtered through 0.22 *μ*m syringe filter.

The extraction was carried out using 5.0 g of powdered roots. It was dissolved in 50 mL of 70% ethanol solution and back-flow for 60 min. After filter and rotary evaporation to no ethanol smell, 50 mL acetic ether was added and extraction was done three times. The extract and washing liquid were combined and filtered and then evaporated to dryness under reduced pressure in a rotary evaporator. The dried extract was dissolved in methanol and diluted to a 5 mL volumetric flask. All sample solutions were filtered through 0.22 *μ*m syringe filter.

### 2.3. Chromatographic Conditions

The HPLC system used was a Shimadzu Nexera X2 UPLC (Kyoto, Japan) chromatograph equipped with a solvent delivery unit (LC-30AD), an autosampler (SIL-30AC), a column oven (CTO-20A), a degasser (DGU-20A5R), and a photodiode array detector (SPD-M20A). Separation was conducted on a Shim-pack XR-ODS column (2.0 × 75 mm, 1.6 *μ*m; Shimadzu Cooperation, Japan). The column temperature was set at 30°C. The mobile phase consisted of water containing 0.1% formic acid (A) and acetonitrile (B). The composition of the mobile phase was 5% (B) for 0–2 min, 5%–10% (B) for 2–4.5 min, 10%–40% (B) for 4.5–11 min, 40%–60% (B) for 11–13 min, 60%–70% (B) for 13-14 min, 70%–80% (B) for 14–16 min, 80%–90% (B) for 16-17 min, 90% (B) for 17–20 min, and it was held for 3 min and then reequilibrated to 5% (B) until the end of the analysis. The flow rate was 0.2 mL/min and the injection volume was 5 *μ*L. The detection wavelengths of all standards and samples were in the UV at 210, 254, and 280 nm.

### 2.4. Method Validation

#### 2.4.1. Linearity

The 4 standard compounds were accurately weighed and dissolved in methanol to prepare stock solutions at a concentration of 1.0 mg/mL. Stock solutions of the compounds were serially diluted to construct calibration curves. The diluted concentrations of compounds were plotted against the peak area on the calibration curves and the linearity was measured from the correlation coefficient.

#### 2.4.2. LOD and LOQ

Blank samples were analyzed in triplicate and the area of the noise peak was calculated as the response. The LOD and LOQ were calculated as LOD = 3.3 × SD/*S* and LOQ = 10 × SD/*S*, where SD is the standard deviation of the response and* S* is the slope of the calibration curve.

#### 2.4.3. Precision

The precision was calculated by analyzing sample extracts containing low and high concentrations of the compounds. The precision was represented by the relative standard deviation (RSD), which was calculated using the equation RSD = (standard deviation/mean) × 100. The precision was measured three times in a single day (intraday precision) and over three consecutive days (interday precision).

#### 2.4.4. Recovery

The accuracy of the method used was evaluated through the recovery test. Both low and high concentrations of the compounds were added to the samples. The recovery was calculated as follows: recovery (%) = ((detected concentration − initial concentration)/spiked concentration) × 100.

## 3. Results and Discussion

### 3.1. Optimization of Chromatographic Conditions

**Figure 2:**
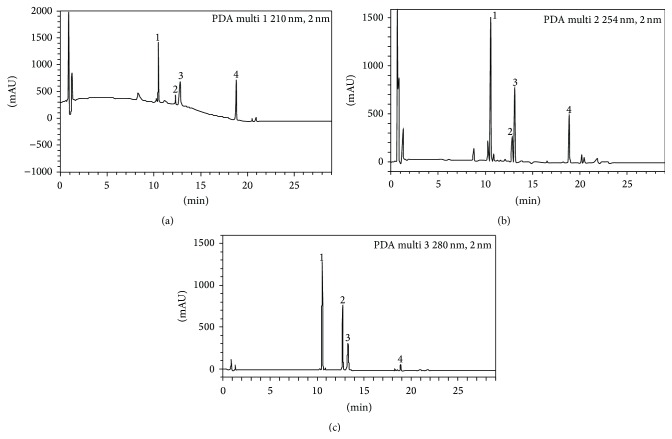
Typical UPLC chromatogram of compounds in* Disporopsis pernyi* (Hua) Diels. (1) Rutin, (2) luteolin, (3) quercetin, and (4) betulinic acid; (a) 210 nm, (b) 254 nm, and (c) 280 nm.

We used PDA detector in this experiment, which could select each wavelength of chromatogram. In our study, we took into account the question that most of the components we studied also have good absorption at wavelength of 350 nm and 370 nm [15, 17]. By comparing the resolution and response at different wavelength, the results showed that the resolution and response of the four components at 350 nm and 370 nm are not as good as the wavelengths 210, 254, and 280 nm we chose. Also, we chose 210 nm to detect betulinic acid for its good response. Combined with the literature reports, methanol-water, acetonitrile-water, and methanol-acetonitrile-water were examined as mobile phase as well as the type (formic acid and glacial acetic acid) and concentration (0.01%, 0.05%, and 0.1%) of the acid. A Shim-pack XR-ODS column was employed for the simultaneous determination of the 4 compounds, as it has been the most frequently used technique in the chemical analysis of herbal medicines by UPLC. The results show that acetonitrile-water has the least interference. Peak resolution and shape of the compounds were considered better indicators when 0.1% formic acid was used as a modifier. Taking peak shape, degree of separation, the symmetrical factor, and other factors into consideration, acetonitrile-0.1% formic acid water solution was determined as gradient elution process. Typical UPLC chromatograms are shown in Figure 2.

### 3.2. Method Validation

#### 3.2.1. Linear Regression, LOD, and LOQ

The linearity of the calibration curve was measured by the correlation coefficient (*r* ^2^), which ranged in value from 0.9992 to 0.9997 for each compound. The LOD and LOQ were 0.137–0.264 *μ*g/mL and 0.456–0.881 *μ*g/mL, respectively (Table 1).

#### 3.2.2. Precision and Recovery

The intra- and interday precision, which were represented by the RSD values, were RSD < 2.0%. The recoveries of the 4 compounds were in the range 96.2%–102.6%, with RSD < 2.0% (Table 2). The results indicate that the developed analytical method was accurate and precise for the analysis of the 4 compounds in* Disporopsis pernyi* (Hua) Diels.

### 3.3. Quantification of the 4 Compounds in* Disporopsis pernyi* (Hua) Diels

The method we developed was applied to determine the 4 compounds in* Disporopsis pernyi* (Hua) Diels successfully. The calculated contents of the four compounds were 5.63 *μ*g/g for rutin, 2.51 *μ*g/g for luteolin, 3.87 *μ*g/g for quercetin, and 2.41 *μ*g/g for betulinic acid. The UPLC method is more simple, duplicate, and effective than HPLC method.

## 4. Conclusions

The UPLC method mentioned here represented an excellent technique for simultaneous determination of rutin, luteolin, quercetin, and betulinic acid in the extract of* Disporopsis pernyi* (Hua) Diels roots, with good sensitivity, precision, and reproducibility. The method gives a good resolution among the four components with the analysis time (25 min). Furthermore, the method can be used as quality control of polyphenolic compounds in* Disporopsis pernyi* (Hua) Diels roots and will play a reference role on the determination of polyphenolic compounds in other medicinal plants or pharmaceutical preparations.

## Figures and Tables

**Table 1:** Regression, correlation coefficient (*r* ^2^), LOD, and LOQ of the 4 compounds in* Disporopsis pernyi *(Hua) Diels.

Compound	UV wavelength	Regression slope	Equation intercept	Linear range (*μ*g/mL)	*r* ^2^	LOD (*μ*g/mL)	LOQ (*μ*g/mL)
Rutin	254 nm	34.63	−23.36	45.10–451.00	0.9992	0.221	0.729
Luteolin	280 nm	45.32	10.61	62.81–439.60	0.9997	0.264	0.879
Quercetin	254 nm	14.47	−24.34	27.58–275.80	0.9993	0.267	0.881
Betulinic acid	210 nm	53.39	8.372	48.34–338.10	0.9992	0.137	0.456

**Table 2:** Recovery of the 4 compounds in *Disporopsis pernyi* (Hua) Diels (*n* = 3).

Compound	Background (*μ*g/mL)	Added (*μ*g/mL)	Found (*μ*g/mL)	Recovery (%)	RSD (%)
Rutin	5.63	2.56	8.32	101.6	1.31
		5.13	10.71	99.5	1.58
		7.70	13.55	101.6	0.89
Luteolin	2.51	1.44	3.94	99.7	1.22
		2.89	5.54	102.6	1.68
		4.34	6.59	96.2	1.06
Quercetin	3.87	1.73	5.42	96.8	1.65
		3.55	7.38	99.5	1.48
		5.19	9.24	102.0	1.37
Betulinic acid	2.41	1.26	3.58	97.5	0.94
		2.52	4.78	97.0	1.89
		3.78	6.25	101.0	1.78
